# Lung histopathological findings in COVID-19 disease – a systematic review

**DOI:** 10.1186/s13027-021-00369-0

**Published:** 2021-05-17

**Authors:** Giuseppe Pannone, Vito Carlo Alberto Caponio, Ilenia Sara De Stefano, Maria Antonietta Ramunno, Mario Meccariello, Alessio Agostinone, Maria Carmela Pedicillo, Giuseppe Troiano, Khrystyna Zhurakivska, Tommaso Cassano, Maria Eleonora Bizzoca, Silvana Papagerakis, Franco Maria Buonaguro, Shailesh Advani, Lorenzo Lo Muzio

**Affiliations:** 1 Anatomic Pathology Unit, Department of Clinic and Experimental Medicine, University of Foggia, 71122 Foggia, Italy; 2 Department of Clinic and Experimental Medicine, University of Foggia, 71122 Foggia, Italy; 3 Department of Surgery, College of Medicine, Health Sciences Center, 107 Wiggins Road, Saskatoon, SK S7N 5E5 Canada; 4 Molecular Biology and Viral Oncology Unit Istituto Nazionale, Tumori IRCCS “Fondazione Pascale”, 80131 Naples, Italy; 5 Georgetown University School of Medicine, Georgetown University, Washington, DC USA

**Keywords:** COVID-19, Histopathology, Lung, Therapy, Systematic review

## Abstract

Since December 2019, the global burden of the COVID-19 pandemic has increased rapidly and has impacted nearly every country in the world, affecting those who are elderly or with underlying comorbidities or immunocompromised states. Aim of this systematic review is to summarize lung histopathological characteristics of COVID-19, not only for diagnostic purpose but also to evaluate changes that can reflect pathophysiological pathways that can inform clinicians of useful treatment strategies. We identified following histopathological changes among our patients:: hyaline membranes; endothelial cells/ interstitial cells involvement; alveolar cells, type I pneumocytes/ type II pneumocytes involvement; interstitial and/ or alveolar edema; evidence of hemorrhage, of inflammatory cells, evidence of microthrombi; evidence of fibrin deposition and of viral infection in the tissue samples.

The scenario with proliferative cell desquamation is typical of Acute Respiratory Distress Syndrome (ARDS) that can be classified as diffuse alveolar damage (DAD) and not DAD-ARDS. The proposed pathological mechanism concerns the role of both innate and adaptive components of the immune system. COVID-19 lethal cases present themselves as a heterogeneous disease, characterized by the different simultaneous presence of different histological findings, which reflect histological phases with corresponding different pathological pathways (epithelial, vascular and fibrotic changes), in the same patient.

**Supplementary Information:**

The online version contains supplementary material available at 10.1186/s13027-021-00369-0.

## Introduction

Since December 2019, the global burden of COVID-19 pandemic has risen rapidly and impacted almost all countries worldwide. Among the wide number of infective diseases, Severe Acute Respiratory Syndrome-Coronavirus 2 (SARS-Cov2) emerged to be a global burden with 123,636,852 cases of infected and 2,721,891 deaths (https://www.ecdc.europa.eu/en/geographical-distribution-2019-ncov-cases). Though majority recovery from this infection, those who are elderly or with underlying comorbidities, frail or immunocompromised states face severe outcomes including Acute Respiratory Distress Syndrome (ARDS), ICU (Intesive Care Unit) admissions, use of ventilator and deaths [1]. Although the majority of patients heal by itself, it is estimated that 13.8% of infected is at risk of severe disease and up to 6.1% can be involved by a critical form of COVID-19 disease, with respiratory failure, septic shock and multiple organ dysfunction or failure (https://www.who.int/docs/default-source/coronaviruse/who-china-joint-mission-on-covid-19-final-report.pdf).

Immune system encompasses a wide range of cellular and chemical messages to deal with pathogens. Both immune system and pathogens characteristics, act in causing damage to the surrounding tissue [2]. In Europe, COVID-19 disease outbreak involved above all Spain and Italy with 182,816 and 168,941 cases respectively, while reporting a higher number of deaths (10.5%; 13.12%). In Italy, in the most affected regions, the lethality rate reached very high peaks with the maximum peak of 18.48% in Lombardy on 17/04/2020, (http://opendatadpc.maps.arcgis.com/apps/opsdashboard/index.html#/b0c68bce2cce478eaac82fe38d4138b1) while the average case fatality rate of the same day was of 13,19% (http://opendatadpc.maps.arcgis.com/apps/opsdashboard/index.html#/b0c68bce2cce478eaac82fe38d4138b1). Government actions, above all social distancing, decreased virus spread, improving the general situation in Europe, meanwhile in USA, India and Brazil the number of new cases is still exponentially growing, with 48,354; 161,736 and 37,017 new cases respectively (https://covid19.who.int/).

Reasons related to this discrepancy are still unclear. Multiple factors may lead to differences in rates of infection and mortality across regions. For e.g. it has been supposed that virus, while infecting Europe, may have mutated and become more aggressive [3–7], while mutation dynamics analysis showed a relative stable genetic sequence and a low rate mutation [8]. In addition, characteristics of populations have to be accounted [4]. Further underlying medical and healthcare system, access to health care facilities and ventilators as well as treatment protocols might have resulted into these differences. At last, different therapy protocols could modify the interaction between virus and host, changing the disease evolution [9–12]. For example, chloroquine and ribavirin were shown to decrease viral infection in vitro, although considering a wide range of adverse effects [13]. In vitro studies might suggest the use of higher doses considering a virus-based approach, with consequences on side effects without evaluating the host-based effect on inflammatory response. Many compounds have shown relevant virus-based efficacy in-vitro models but with several limitations on clinical reproducibly. For example, some of them requires high EC_50_/C_max_ ratios at clinically relevant dosages, with consequences of side effects like immunosuppression [14]. Failing of some therapies furthermore, can be associated to a delay on drug administration in relation to symptoms occurrence or lack of availability to the masses or differences in efficacy and effectiveness in specialized subgroups of population [14, 15].

SARS-Cov2 belongs to the family of coronavirus together with Severe Acute Respiratory Syndrome (SARS) and Middle East Respiratory Syndrome (MERS). Little is known of the impact of SARS-CoV2 on lung tissue damage. The aim of this work is to critically review histopathological findings of COVID-19, focusing on pathological mechanisms, in order to suggest a target therapy approach, virus or host-based.

## Methods

### Protocol and registration

This systematic review has been carried out following the “Preferred Reporting Items for Systematic Reviews and Meta Analyses” (PRISMA) guidelines [16]. Prospectively, the protocol for this systematic review has been registered on International prospective register of systematic reviews (PROSPERO) with the following registration code: CRD42020182279.

### PICO question

PICO (Population, Intervention, Comparison and Outcome) question was relevant to assess: Population (such as patients diagnosed of COVID-19 disease), Intervention (undergoing histopathological examination), Control (not applicable) and Outcome (histopathological changes related to SARS-Cov-2 infection).

The formulation of the PICO question was as follows: what are the histopathological changes in lungs of patients affected by SARS-COV-2 infection during COVID-19 disease?

PICO highlighted limitation of the current study, since a comparison control group was missing in the included studies (case reports/case series).

#### Eligibility criteria

We took into consideration studies written and published in English language only. Only clinical studies were included, reporting, in both prospective and retrospective case reports, case-series and cohort studies, the histopathological findings in lungs of patients with confirmed death of COVID-19 disease because of SARS-Cov2 infection. Studies to be included had to report an accurate description of histopathological changes/images or reported through their autopsy reports.

At last, studies performed in vitro, in animal models or lacking evidence of SARS-Cov2 infection were excluded from this systematic review.

#### Information sources and search strategy

Two authors (I. S. D. S. and M. A. R.) independently carried out an online bibliographic search through PubMed and Web of Science. This search started on April 1st 2020 and was continued every day before the last search on 1st June 2020, because of the high number of articles published daily about COVID-19 disease. MeSH terms and free text words were combined using Boolean operators (AND, OR). The following search string was used and input in the search bars of the previous reported databases: (COVID-19 OR SARS-Cov2) AND (histopathological finding OR paraffin OR pathology OR histopathology OR autopsy).

#### Screening, study selection and data collection process

All searches from our databases were put in excel sheets designed for title and abstract screening. Screening of target studies was performed independently by two authors (I. S. D. S. and M. A. R.) through all the resulting research articles. We calculated Cohen’s kappa to calculate interrater reliability in screening process between two reviewers. A score of 0.71 represents good agreement between reviewers. In case of disagreement, during the screening and selection process, a third reviewer (A. A.) took the final decision through mutual consensus in a joint discussion Studies meeting the previous reported inclusion criteria, proceeded to full text review and data abstraction. M. M. and A. A. reviewed and completed the full text abstraction. Following information were retrieved from each article: Author’s first name, Year of publication, Country, number of patients, mean age, sex, comorbidities, hyaline membranes, comorbidities, evidence of endothelial cells / interstitial cells involvement, presence or absence alveolar cells, type I pneumocytes/ type II pneumocytes involvement, evidence of inflammatory cells, sampling methods for histopathological examination, detection methods, evidence of microthrombi, evidence of hemorrhage, fibrin deposition, interstitial/alveolar edema and viral detection method within the cells. A. A. and M. M performed the data extraction.

#### Risk of bias assessment

Included studies underwent quality check and risk of bias assessment. This qualitative analysis was performed according Murad’s quality checklist of case series and case report [17]. As reported, the scale consists of four parameters, to evaluate the a) patient selection; b) exposure ascertainment; c) causality and d) reporting. Each section contains one to four question to be addressed. As it is suggested we performed an overall judgement about methodological quality since questions 4, 5 and 6 are mostly relevant to cases of adverse drug events. Each requested field will be considered as adequate, inadequate or not evaluable. The table showing this tool for evaluating the methodological quality of case reports and case series, is reported in the original manuscript [17].

## Results

### Study selection

**Fig. 1:**
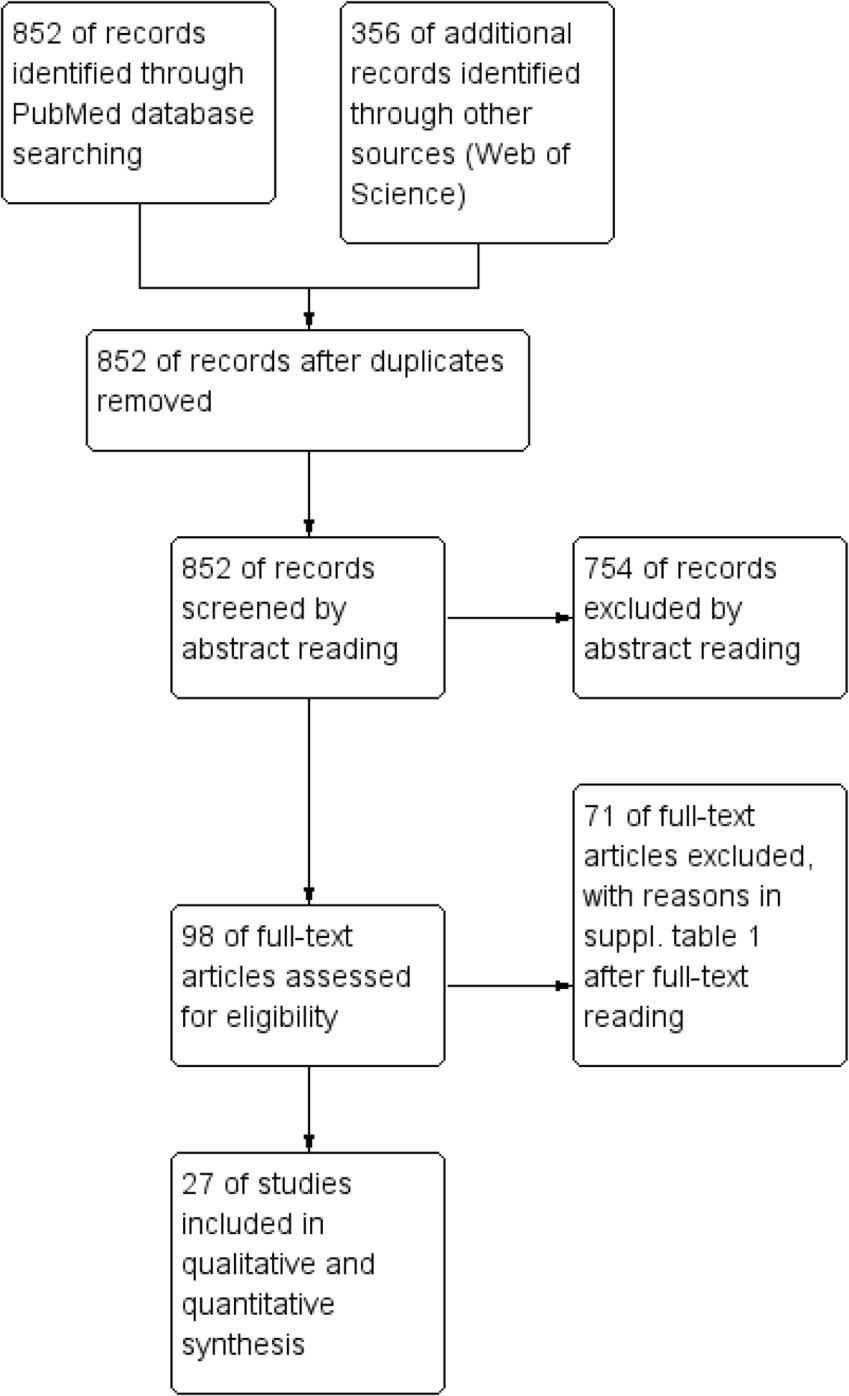
PRISMA Flowchart of selection process

Last search was performed on 01/06/2020, reporting a total of 852 records on PubMed database and 356 on Web of Science. These records were screened only by title and abstract and a total number of 98 articles passed the first selection process. Resulting records underwent full-text reading to meet the inclusion criteria. After this last step, a total of 27 articles fulfilled the inclusion criteria and were included in the quality and synthesis. Prisma Flowchart summarizing the selection process is reported in Fig. 1, studies and reasons for exclusion are collected in supplemental Table 1. The level of agreement between reviewers in the selection process was good since the K-agreement statistic reported a value of 0.71.

### Characteristics of included studies and methodological quality assessment

A total number of 196 patients, who died of COVID-19 disease, underwent surgical specimen resection through biopsy (Yao et al. [18]; Xu et al. [19]; Varga et al. [20]; Harkin et al. [21]; Li et al. [22]; Shao et al. [23]; Pernazza et al. [24]; Tian et al. [25]; Tian et al. [26]; Cai et al. [27] and Zeng et al. [28]) or post-mortem autopsy (Fox et al. [29]; Lacy et al. [30]; Menter et al. [31]; Barton et al. [32]; Karami et al. [33]; Magro et al. [34]; Lax et al. [35]; Adachi et al. [36]; Yan et al. [37]; Ackermann et al. [38]; Schaller et al. [39]; Martines et al. [40]; Buja et al. [41]; Sekulic et al. [42]; Aguiar et al. [43] and Konopka et al. [44]) to assess histopathological changes in lung parenchyma. Most of studies were performed in China [18, 19, 22, 23, 25–28, 30], together with USA [21, 29, 32, 34, 37, 40–42, 44]. Seven studies were performed in Europe [20, 24, 31, 35, 38, 39, 43], while remaining ones were performed in Japan [36] and Iran [33]. Of these 196 patients, 66 were females,. Comorbidities were reported in most of patients. Only one patient reported no comorbidities [23] meanwhile six studies did not reported [18, 19, 21, 33, 36, 38]. In general, most commonly reported systemic diseases were diabetes, obesity, hyperlipidemia, hypertension and cardiovascular disease. Some patients reported concomitant lung diseases, such as lung adenocarcinoma [24, 26, 27] or Chronic Obstructive Pulmonary Disease (COPD) [22, 27, 31]. Population characteristics are summarized in Table 1.

**Table 1:** List of included studies and data extraction

Author (year)	Country	Patients (n)	Mean age (years)	Sex (M/F)	Comorbidities	Hyaline membranes	Endothelial cells alteration (ECA), interstitial cells involvement (ICI)	Alveolar cells: Type I pneumocytes/ Type II pneumocytes involvement	Hemorrhage	Sampling methods	Detection methods	(Micro)thrombi	Inflammatory cells	Fibrin deposits	Interstitial/ alveolar edema	Viral detection methods
Li et al. (2020) [22]	China	69	51	M/F 40/29	Diabetes (8), hypertension (17), coronary disease (4), chronic liver disease (7), chronic respiratory system disease (7), malignancy (1)	Reported (2)	ICI	DAD, pneumocyte hyperplasia, desquamation of pneumocytes	NA	Biopsy	H&E, Masson’s trichrome stain, IHC, EM	NA	CD68+ macrophages, neutrophils, CD4 + and CD8+ T cells	NA	Focal exudative edema	Yes, EM, ISH
Buja et al. (2020) [41]	USA	23	55	M/F 12/7	Obesity, hypertension, myotonic dystrophy, type II diabetes	Absent	ICI	Type I,II pneumocytes hyperplasia	Reported	Autopsy	RT-PCR	Formation of microthrombi in arterioles	Neutrophils, lymphocytes, macrophages	Intra-vascular and intra-vascular edema	Reported	Yes, EM
Menter et al. (2020) [45]	Switzerland	21	76	M/F 17/4	Hypertension (21), cardiovascular disease (15), diabetes mellitus (7), COPD (3), chronic neurological conditions (5), malignancy (3), chronic liver disease (2), chronic kidney disease (4), acquired immunosuppression (1)	Reported	Capillary congestion (21), vasculitis (1)	Syncytial cells of pneumocytes II origin (11), DAD (8)	Reported (3)	Autopsy	IHC for fibrin, 360x, H&E, IHC	Microthrombi of alveolar capillaries (5)	Prominent lymphoid infiltrate (3)	NA	Interstitial edema	Yes, SARS-CoV-2 specific RT–qPCR, viral genome detected with the TaqMan 2019-nCoV Assay Kit v1 (Thermo Fisher Scientific) targeting three different viral genomic regions (ORFab1,S Protein, N Protein)
Lax et al. (2020) [35]	Austria	11	81	M/F 8/3	Diabetes mellitus, cardiovascular disease	Reported (11)	ICI, fibroblast proliferation (9)	Pneumocytes proliferation (9)	Reported (8)	Resection	H&E	Reported (11)	Macrophages, lymphocytes, plasma cells, neutrophils	Reported	Reported (10)	Yes, RT-PCR results positive in the right and left bronchus ´10/10)
Fox et al. (2020) [29]	USA	10	61	NA	At least one comorbidity, the most common: hypertension, type II diabetes, obesity	Reported (2)	ICI, CD61+ megakaryocytes with nuclear hypercromasia and atypia	DAD (10), desquamation	Foci of hemorrhage (9)	Autopsy	H&E	Reported (11)	CD4+ and CD8+ lymphocytes	Reported	Reported (2)	DRAQS and SYTO RNASelect fluorescent staining
Schaller et al. (2020) [39]	Germany	10	79	M/F 7/3	Hypertension, hypothyroidism, diabetes	Reported	ICI, fibroblastic cells	Pneumocytes hyperplasia	NA	Autopsy	IHC	Microthrombireported	Lymphocytes	Reported	Alveolar edema	Yes, RT-PCR, IHC
Martines et al. (2020) [40]	USA	8	74	M/F 4/4	Hypertension, chronic kidney disease, diabetes, obesity	NA	NA	Type II pneumocytes hyperplasia	Reported	Autopsy	IHC, H&E	Microthrombi observed	Macrophages, neutrophils, leukocytes	Reported	Alveolar edema	Yes, IHC (rabbit polyclonal antibody), RT-PCR
Ackermann et al. (2020) [46]	Germany	7	73	M/F 5/2	NA	NA	ECA	Type II pneumocytes hyperplasia	Reported	Autopsy	IHC, scanner election microscopy	Fibrin thrombi	T lymphocytes	Intra-alveolar fibrin	Mild interstitial edema	No
Cai et al. (2020) [27]	China	7	60	M/F 5/2	Interstitial lung disease (1), coronary atherosclerosis (3), chronic obstructive pulmonary disease (2), hyperlipidemia	Absent (1)	ICI, fibrous connective tissue proliferation	No evident pneumocytes hyperplasia (1)	NA	Biopsy	H&E	NA	Plasma cells and macrophages (1)	NA	NA	No
Magro et al. (2020) [34]	USA	5	55	M/F 3/2	Coronary heart disease, diabetes, hepatitis C	Reported	ICI	Type II	Reported	Biopsy	IHC,RT-PCR	Reported	AP and LP of complement	Reported	Reported	Yes, using NUANCE software
Tian et al. (2020) [47]	China	4	73	M/F 3/1	Chronic lymphocytic leukemia, cirrhosis, variceal rupture bleeding, diabetes, hypertension, status post renal transplantation for 3 months	Reported (3)	ICI, fibrinoid necrosis of the small vessels	DAD, type II hyperplasia, syncytial giant cells formation pneumocytes	Reported	Biopsy	H&E, IHC	NA	scanty inflammatory cells	Reported	NA	Yes, RT-PCR positive only in case 2
Varga et al. (2020) [20]	Switzerland	3	66	M7F 2/1	Coronary heart disease, hypertension, diabetes, obesity	NA	ECA	NA	NA	Biopsy	IHC	NA	Mononuclear cells, apoptotic bodies	NA	Reported	Yes, EM in kidney and small bowel tissue but no obvious viral particles in the lung tissue
Barton et al. (2020) [48]	USA	2	60	M/F 2/0	Hypertension, remote deep veins thrombosis, myotonic dystrophy	Reported	Congestion of septal capillaries	DAD in the acute stage (only in one case)	NA	Autopsy	IHC, H&E	Microthrombi reported	CD3+ T lymphocytes, rare CD20+ lymphocytes, CD8+ T lymphocytes, macrophages, non appreciated neutrophils oreosinophils	NA	Reported	Bilateral lung parenchymal swabs positive only in one case
Tian et al. (2020) [26]	China	2	79	M/F 1/1	Lung adenocarcinoma	Not prominent	ICI, proliferating fibroblasts 1 case)	NA, type II pneumocytes hyperplasia (1 case)	NA	Autopsy	H&E	NA	Mononuclear inflammatory cells	Focal fibrin exudates	Alveolar edema	No
Sekulic et al. (2020) [42]	USA	2	68	M/F 2/0	Diabetes mellitus relates renal disease … left ventricular hypertrophy, atherosclerotic coronary artery disease, hypertension, congestive splenomegaly, sinusoidal congestion of the liver	Reported (2)	fibroblastic proliferation	DAD, scattered multinucleated giant cells, squamous metaplasia features of type II pneumocytes viral infection	NA	Autopsy	H&E, EM	NA	Relative paucity of chronic inflammatory cells	Intra-alveolar fibrin deposition	Interstitial edema	Yes, PCR (2)
Yao et al. (2020) [18]	China	1	78	M/F 0/1	NA	Reported	ICI	Type II; DAD with desquamation or proliferative type II cells	NA	Biopsy	PCR(X3), IHC, H&E	Hyaline thrombi in micro vessels	Macrophages, CD8+ and CD4+ T cells, CD20+ cells	Reported	No pulmonary edema	Yes, EM, IHC with monoclonal anti-nucleoprotein antibody
Lacy et al. (2020) [49]	China	1	58	M/F 0/1	Type II diabetes, obesity, hypertension	Reported	ICI, no fibroblastic foci	NA, pneumocyte hyperplasia, no viral inclusion or specific cytopathic changes,focal multinucleated cells	Reported	Autopsy	RT-PCR	NA	Macrophages	Reported	Diffuse proteinaceous edema	No viral inclusion or cytopathic changes identified
Xu et al. (2020) [19]	China	1	50	M/F 1/0	NA	Reported	NA	Desquamation, early ARDS, atypical enlarges pneumocytes with large nuclei, amphophilic granular cytoplasm and prominent nucleoli. No obvious intranuclear or intracytoplasmic viral inclusion identified	NA	Biopsy	NA	NA	Lymphocytes	NA	Reported	No obvious viral inclusion identified
Karami et al. (2020) [33]	Iran	1	27	M/F 0/1	NA	Reported	NA	Pneumocyte proliferation, multinucleation and nuclear atypia, metaplastic changes	NA	Autopsy	NA	NA	Lymphocytes and macrophages	NA	NA	Yes, RT-PCR confirmed SARS-CoV-2 infection in the lungs
Harkin et al. (2020) [21]	USA	1	34	M/F 1/0	NA	Absent	NA	Type II pneumocytes hyperplasia	NA	Autopsy	RT-PCR	NA	Lymphocytes, hystiocytes	Intra-alveolar fibrin deposits	NA	Yes, RT-PCR positive in BAL
Shao et al. (2020) [50]	China	1	65	M/F 1/0	Absent	Reported	ICI, proliferating fibroblasts, dilated pulmonary capillaries	DAD, type II pneumocyte multinucleation, hyperplasia and increased nuclear size, hypercromasia, hyperplasia of alveolarepithelial cells	NA	Biopsy	IHC, GMS	Reported (11)	Lymphocytes, neutrophils, macrophages	Intra-alveolar fibrin exudate	NA	No, IHC staining for anti-SARS-Cov-2 N Protein was negative
Adachi et al. (2020) [36]	Japan	1	84	M/F 0/1	NA	Reported	ICI, vascularcongestion	Type II pneumocytes hyperplasia, squamous metaplasia, desquamation	Intra-alveolar hemorrhage	Autopsy	H&E, IHC	NA	Plasma cells	NA	NA	Yes, rabbit polyclonal antibodies
Yan et al. (2020) [37]	USA	1	44	M/F 0/1	Obesity	Reported	Non necrotizing lymphocytic vasculitis	DAD, desquamation, pneumocytes with ample cytoplasm and enlarged nuclei	NA	Autopsy	EM	Not identified	Lymphocytes	Fibrin aggregates within blood vessels	NA	No
Pernazza et al. (2020) [51]	Italy	1	61	M/F 1/0	Lung adenocarcinoma	Absent	ICI	Pneumocytes desquamation, reactive hyperplasia with focal nuclear inclusion	Diffuse hemorrhage	Biopsy	H&E, IHC	NA	inflammatory infiltrate mainly composed by cytotoxic (CD8+) T lymphocytes, neutrophilic vascular margination, macrophages	Scanty fibrin deposited on the alveolar surface	Interstitial edema	No
Aguiar et al. (2020) [43]	Switzerland	1	31	M/F 0/1	Obesity, hypertension, myotonic dystrophy, type II diabetes	Reported	Vascular stasis, reported megakaryocytes	DAD, type II pneumocytes hyperplasia neither viral inclusion nor giant multinucleated giant cells	Hemorrhagic edema, alveolar hemorrhage	Autopsy	H&E, IHC	Absence of hyaline thrombi in microvessels	intra-alveolar macrophages, PMN, T (CD3+) lymphocytes	Intra-alveolar fibrin deposition	Reported	Yes, rRT-PCR in the lower respiratory tract
Konopka et al. (2020) [44]	USA	1	37	M/F 1/0	Asthma, type II diabetes	Scattered hyaline membranes	ECA	DAD, mucous plugs, mucous glands, hyperplasia, type II pneumocytes hyperplasia	NA	Autopsy	H&E	Rare within small vessels	scattered neutrophils	Fibrin-airspace exudate	Reported	No
Zeng et al. (2020) [28]	China	1	55	M/F 0/1	Pulmonary node	Not observed	ICI	Type II pneumocytes hyperplasia	NA	Lobectomy	H&E	NA	Monocytes, T (CD3+), B (CD20 + and PAX5+) lymphocytes, (MUM1+) plasma cells, CD4+ helper and CD8+ cytotoxic lymphocytes, natural killer (CD56+) macrophages, (CD163+) M2 macrophages	Not observed	Reported	Yes, PCR, in situ hybridization technology

### Main histopathological reported findings of Covid-19 related lung disease

Main histopathological changes of Covid-19 related lung disease were reported as presence of following histopathological changes:: hyaline membranes; endothelial cells / interstitial cells involvement; alveolar cells, type I pneumocytes/ type II pneumocytes involvement; interstitial and/or alveolar edema; evidence of hemorrhage; evidence of inflammatory cells; evidence of microthrombi; evidence of fibrin deposition and evidence of viral infection in the tissue sample.

#### Hyaline membranes

Hyaline membranes were reported in 19 (21, 22, 25, 26, 28, 32–40, 42, 45–47) out of 27 included studies representing a histological report in almost 80% of studies performed on lethal cases of COVID-19 disease. In one study, including two patients, hyaline membranes were reported to be not prominent [26] or scattered [44]. Therefore, while three studies did not report any information about this parameter [20, 38, 40], hyaline membranes were reported in 19/24 of included studies representing a histological report in almost 80% of studies performed on lethal cases of COVID-19 disease.

#### Interstitial cell involvement

Interstitial cell involvement was commonly reported in seven studies [18, 22, 28, 30, 34, 41, 42]. Interstitial cell involvement, associated with fibroblast proliferation, was reported in Tian et al. and Lax et al. studies [26, 35]. Schaller et al. also reported interstitial cell involvement with fibroblast proliferation [39], together with Sekulic et al. [42]. Cai et al. reported interstitial cell involvement and fibrous connective tissue proliferation [27]. Interstitial cell involvement together with CD61+ megakaryocytes with hyperchromatic nuclei and atypia was reported in Fox et al. study [29]. Endothelial cell alteration (ECA) was reported by Menter et al. as capillary congestion in all included patients (twenty-one), meanwhile only one patient reported vasculitis [31]. Congestion, specifically of septal capillaries, was reported in two patients in Barton et al. [32]. Ackermann et al., Varga et al. and Konopka et al. reported generic ECA [20, 38, 44], meanwhile Yan et al. reported non-necrotizing lymphocytic vasculitis. At last, Aguiar et al. reported vascular stasis with megakaryocytes [43].

Both, interstitial cell involvement and ECA, were reported in three studies [23, 25, 36]. In Xu et al., Karamy et al., Harkin et al. and Martines et al. studies, interstitial cell involvement and ECA were not investigated [21, 33, 40].

A wide spectrum of epithelial alterations was reported involving alveolar cells, above all type II pneumocytes. Specifically, some studies attributed hyperplasia to type II pneumocytes, describing DAD with desquamation of proliferative cells [18, 19, 22, 24, 28, 29, 36, 37, 43, 44] and syncytial cell formation [25, 31]. Karami et al., Adachi et al. and Sekulic et al. reported metaplastic changes associated to multinucleation and nuclear atypia [33, 36, 42]. Varga et al. did not report any information [20]; Cai et al. stated no evidence of pneumocyte hyperplasia [27]. In total 19 studies reported epithelial changes consistent with epithelial histological pattern of acute lung injury.

#### Alveolar edema

Another characteristic of lung tissue damage is the interstitial or alveolar edema. Well defined interstitial edema was reported in four studies [24, 31, 38, 42]. Lacy et al. described diffuse proteinaceous edema [30], meanwhile most of authors reported generic edema [19, 20, 22, 28, 29, 32, 34, 35, 41, 43, 44]. Alveolar edema was found in few reports [26, 39, 40]. In Yao et al. record there was no pulmonary edema [18]. These features were not evaluated in the remaining studies [21, 23, 25, 27, 33, 36, 37].

#### Hemorrhage

. Twelve studies reported events of hemorrhage [25, 30, 31, 34, 35, 38, 40, 41, 43], with different severity ranging from intra-alveolar hemorrhage [36], focal [29] to diffuse [24]. By evaluating studies that reported these events, almost 45% of studies with lethal cases of COVID-19 disease presented this histological finding.

#### Inflammatory cells

Macrophages were the most abundant cell-type, reported in 13 over 27 studies [18, 21, 23, 24, 27, 28, 30, 32, 33, 35, 40, 41, 43]. Lymphocytes were the second most abundant kind of inflammatory cell infiltration [19, 21, 23, 28, 29, 33, 35, 37, 39, 41, 43]. In Ackermann et al. report there was evidence of T-cell infiltration [38], while Yao et al. describe both T- and B-cell infiltration. Patients displayed also plasma cells [27, 35, 36] and neutrophil infiltration [23, 35, 40, 41, 44]. A nonspecific lymphoid infiltrate was indicated in four studies [20, 26, 31, 34]. Moreover, some studies reported a deeper analysis of inflammatory cell infiltration. Yao et al. highlight the presence of macrophages, CD8+ and CD4+ T cells and CD20+ B cells [18], meanwhile in Barton et al. samples there was evidence of CD3+ T lymphocytes, rare CD20+ B lymphocytes, CD8+ T lymphocytes and CD4+ T cells, macrophages, while neutrophils or eosinophils were not appreciated [32]. Li et al. also reported CD68+ macrophages, neutrophils, CD4+ and CD8+ T cells [22]. Inflammatory infiltrate mainly composed by cytotoxic (CD8+) T lymphocytes, neutrophilic vascular margination and macrophages were descripted by Pernazza et al. [24]. Zeng et al. performed a detailed analysis of lymphoid infiltrate, demonstrating evidence of monocytes, T (CD3+), B (CD20+ and PAX5+) lymphocytes, (MUM1+) plasmacells, CD4+ helper and CD8+ cytotoxic lymphocytes, natural killer (CD56+), (CD68+) macrophages and (CD163+) M2 macrophages.

At last, Tian et al. [25] and Sekulic et al. [42] were the only one reporting scanty inflammatory cells.

#### Microthrombi

Microthrombi were reported in twelve studies [18, 23, 29, 31, 32, 34, 35, 38–41, 44]. Yao et al. descripted hyaline thrombi in microvessels, while were found as fibrin thrombi in Ackermann et al. study [18, 38]. Menter et al. reported evidence of microthrombi of alveolar capillaries [31].

#### Fibrin deposits

Another important histopathological change was fibrin deposits. These were descripted mainly as intra-alveolar [21, 23, 37, 38, 42, 43], in addition Buja et al. reported the contemporary presence of extravascular deposits [41]. In one study, scanty fibrin deposits were reported on the alveolar surface [24].

**Fig. 2:**
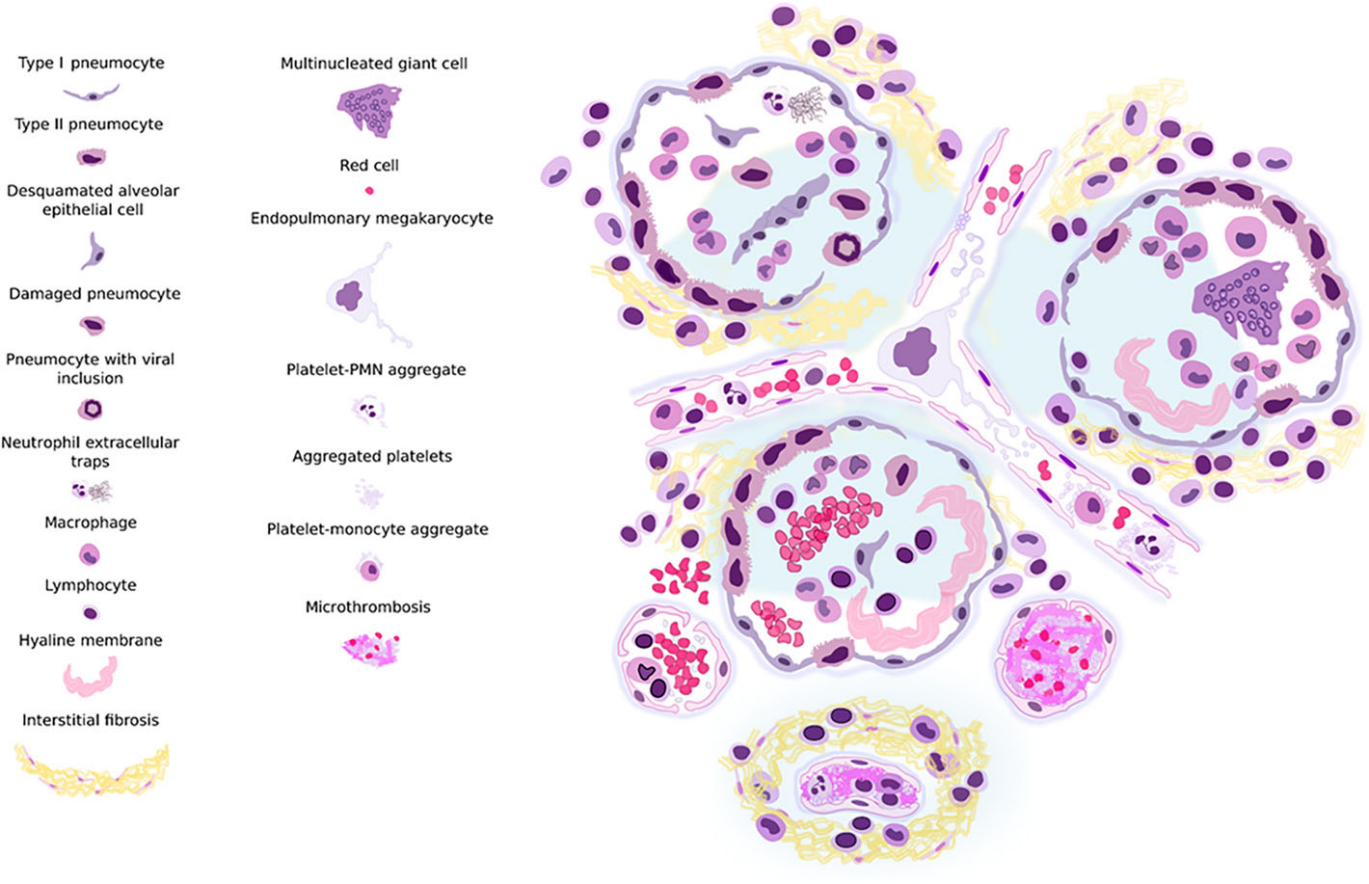
Summary of histopathological features occurring in fatal cases of COVID-19 lung injury. Findings of epithelial, vascular and fibrotic pattern are represented. Note that epithelial damage like viral cytopathic changes, desquamation and reactive hyperplasia of type II pneumocytes, hyaline membrane formation and interstitial inflammatory response have been frequently observed together with findings of vascular damage like capillary congestion, intracapillary microthombosis, alveolar hemorrhage, interstitial and intra-alveolar edema. Furthermore, interstitial fibrous changes, occurring separately or in combination with epithelial and/or vascular injury in a subgroup of patients, are shown. For further details see descriptive text

Representation of main histological features of COVID-19 in lung tissue pathology are collected in Fig. 2.

#### SARS-Cov2 in lung tissue: Histopathological examination

Last evaluated parameter was the confirmation of SARS-Cov2 in the lung tissue. This exploration was performed through different techniques. Yao et al. carried out electronic microscope investigation together with IHC with monoclonal anti-nucleoprotein antibody [18]. In Xu et al. and Lacy et al. studies there were no viral inclusion or cytopathic changes [19, 30]. Menter et al. investigated through SARS-CoV2 specific qRT-PCR, viral genome detected with the TaqMan 2019-nCov Assay Kit v1 (Thermo Fisher Scientific) targeting three different viral genomic regions (ORFab1, S Protein, N Protein) [31]. Barton performed the inspection on bilateral lung parenchymal swab [32]. qRT-PCR analysis was the most common methodology [21, 25, 28, 33, 35, 39, 40, 42, 43], followed by IHC and FISH [18, 22, 23, 28, 29, 39, 40]. Buja et al. investigated through electronic microscope [41].

#### Risk of bias assessment

Results from quality and risk of bias assessment are summarized in Table 2. Briefly, only four studies fulfilled the quality checklist [22, 29, 34, 35]. Only one study [21] failed in “domains - leading explanatory questions” since the aim of the study was to report the delay in COVID-19 disease diagnosis. “Selection - Does the patient(s) represent(s) the whole experience of the investigator (center) or is the selection method unclear to the extent that other patients with similar presentation may not have been reported?” checklist resulted unclear in most of studies, because the patient selection method was unclear [18–21, 23, 26–28, 30–33, 36, 37, 40–44]. In general, overall quality was satisfactory in all included studies, but Varga et al. [20], where exposure to SARS-Cov2 was not ascertained, together with Ackermann et al. study [38]. Konopka et al. reported a positive test results for SARS-Cov2 without method and material details. A complete report of quality checklist is reported in Table 2.

**Table 2:** Qualitative evaluation of included study to assess the risk of bias

Author	Country	Domains	Selection	Ascertainment (1) (2)		Casuality	Reporting
Yao et Al.	China	Ascertained	Unclear	Ascertained	Ascertained	Ascertained	Ascertained
Lacy et Al.	China	Ascertained	Unclear	Ascertained	Ascertained	Ascertained	Ascertained
Menter et al.	Switzerland	Ascertained	Unclear	Ascertained	Ascertained	Ascertained	Ascertained
Barton et Al	USA	Ascertained	Unclear	Ascertained	Ascertained	Ascertained	Ascertained
Xu et al.	China	Ascertained	Unclear	Ascertained	Ascertained	Ascertained	Ascertained
Karami et Al.	Iran	Ascertained	Unclear	Ascertained	Ascertained	Ascertained	Ascertained
Varga et al.	Switzerland	Ascertained	Unclear	Unclear	Ascertained	Unclear	Ascertained
Magro et al.	USA	Ascertained	Ascertained	Ascertained	Ascertained	Ascertained	Ascertained
Pernazza et al.	Italy	Ascertained	Ascertained	Ascertained	Ascertained	Unclear	Ascertained
Ackermann et al.	Belgium	Ascertained	Ascertained	Unclear	Ascertained	Ascertained	Ascertained
Harkin et al.	USA	Unclear	Unclear	Ascertained	Ascertained	Ascertained	Ascertained
Fox et al.	USA	Ascertained	Ascertained	Ascertained	Ascertained	Ascertained	Ascertained
Tian et al.	China	Ascertained	Ascertained	Ascertained	Ascertained	Unclear	Ascertained
Tian et al.	China	Ascertained	Unclear	Ascertained	Ascertained	Unclear	Ascertained
Shao et al.	China	Ascertained	Unclear	Ascertained	Ascertained	Ascertained	Ascertained
Buja et al.	USA	Ascertained	Unclear	Ascertained	Ascertained	Ascertained	Ascertained
Li et al.	China	Ascertained	Ascertained	Ascertained	Ascertained	Ascertained	Ascertained
Yan et al.	USA	Ascertained	Unclear	Ascertained	Ascertained	Ascertained	Ascertained
Adachi et al.	Japan	Ascertained	Unclear	Ascertained	Ascertained	Ascertained	Ascertained
Schaller et al.	Germany	Ascertained	Ascertained	Ascertained	Ascertained	Ascertained	Unclear
Martines et al.	USA	Ascertained	Unclear	Ascertained	Ascertained	Ascertained	Ascertained
Lax et al.	Austrai	Ascertained	Ascertained	Ascertained	Ascertained	Ascertained	Ascertained
Cai et al.	China	Ascertained	Unclear	Ascertained	Ascertained	Ascertained	Ascertained
Sekulic et al.	USA	Ascertained	Unclear	Ascertained	Ascertained	Ascertained	Ascertained
Aguiar et al.	Switzerland	Ascertained	Unclear	Ascertained	Ascertained	Ascertained	Ascertained
Konopka et al.	USA	Ascertained	Unclear	Unclear	Ascertained	Ascertained	Ascertained
Zeng et al.	China	Ascertained	Unclear	Ascertained	Ascertained	Ascertained	Ascertained

## Discussion

Despite the high lethality and mortality of SARS-CoV-2 infection, very limited research exists on histological aspects of COVID-19 disease in the literature. Some histological aspects have been described because there was no awareness of the infection and patients had been operated on for other reasons [21, 24]. In most of cases, autopsy procedures were not performed because of high standard security protocols needed, since, as reported by Zhou et al., viral shedding continues until death among people who passed away from COVID [52]. However, histopathology is still considered the gold standard procedure to assess for pathological changes across a broad range of diseases [53]. In this scenario, the aim of this systematic review is to provide summarizing lung histopathological characteristics of COVID-19 disease, not only for diagnostic purpose but also to evaluate changes that can reflect physio-pathological pathways that can inform clinicians of useful treatment strategies.

From this systematic review, lung injury itself reflects histopathological alterations including alveolar and interstitial alterations, type II pneumocyte hyperplasia and cytological atypia, with hyaline membrane formation, while only around 20% of studies with lethal cases of COVID-19 disease showed fibroblast proliferation. Generally, this scenario is typical of ARDS, with desquamation of proliferative cells and follows the clinical diagnostic criteria of the Berlin definition [54]. Histologically, ARDS can be classified as DAD and non-DAD-ARDS [55]. Both Guerin and Thille showed a mean association of clinical ARDS with DAD, respectively in 58 and 56% of patients, with an increase of this association up to 69% in patients with severe forms of ARDS [56, 57].

Although many studies shared histopathological evidence of DAD, only nine studies reported diagnostic clinical criteria of ARDS, for which patients died [19, 22, 29, 33, 34, 37, 38, 41, 44]. Different studies [19, 58, 59] suggest the use of corticosteroid to prevent ARDS evolution. Specifically in COVID-19 disease; Raju et al. in their systematic review and meta-analysis of registered trials reported promising results of corticosteroids in the treatment of severe form of COVID-19 disease, although highlighting limitations [60]. Sarma et al. performed a similar study by investigating the use of steroids in the management of diverse forms of severity of the disease. In this meta-analysis emerged that patients with severe disease benefitted of steroids therapy, reducing mortality and the need of assisted ventilation, while no improvements were observed in patients with mild forms of COVID-19 [61]. Moreover, in a meta-analysis of randomized study including 1703 patients, low-dosage corticosteroids improved survival in hospitalized patients needing respiratory assistance [62]. Xu et al. reported common histopathological alteration of ARDS, such as hyaline membranes and pneumocytes desquamation with cellular changes. Same alterations were reported by Li et al. [22] who also highlight how pro-inflammatory cytokines were higher during disease worsening and suggest the use of therapy targeting those cytokines.

Moreover, Magro et al. suggest that COVID-19 disease differs from typical ARDS at histopathological level. Patients included in their study reported changes to the alveolar capillaries, signs of thrombotic microvascular injury. This phenomenon leads to the activation of clotting pathway with consequent fibrin deposition [34]. Vascular changes were also found in Ackermann et al. study [38]. In this scenario, although COVID-19 disease seems to cause lung injury through an epithelial pattern, another study evaluating patient level data lung changes and symptoms, defined three different patterns of lung damage. In Polak et al. review of 129 patients, 110 reported lung epithelial damage, 76 patients vascular damage and 28 fibrotic changes. These patterns were mixed in 47 patients with 32 patients reporting overlapping epithelial and vascular damage. Furthermore, epithelial damage was consequence of host viral response in the early phase of the disease, followed by host inflammatory response and hypercoagulation leading to a vascular pattern, ending in a fibrosis of pulmonary tissue in the 22% of patients. Although these interesting results, patterns of damage can overlap in some patients and can be prominent in different stages of COVID-19 disease [63]. From our systematic review, vascular pattern of interstitial lung injury, mainly characterized by microthrombi and proteinaceous and fibrinous exudate associated to edema is often associated to the dominant epithelial damage, at the base of DAD. Therefore, ARDS in COVID-19 disease should not be intended as independent from DAD, but ARDS in these patients reflects the common denominator of DAD with the addition of vascular damage. This vascular damage is more common in COVID-19 lung disease, compared to other kinds of different origins ARDS.

The vascular endothelium is an active paracrine, endocrine and autocrine organ that is indispensable for the regulation of vascular tone and the maintenance of vascular homeostasis [64]. Endothelial dysfunction is the main cause of microcirculation alterations by moving the vascular balance more towards vasoconstriction with subsequent organ ischemia, inflammation with associated tissue edema and procoagulant state [65]. The SARS-CoV-2 coronavirus accesses host cells through the binding of its spike glycoprotein with the angiotensin 2 converting enzyme, sialic acid receptor, serine protease 2 transmembrane and extracellular matrix inducer metalloproteinase CD147; cathepsin B and L also participate in the entry of the virus. All these factors are expressed in endothelial cells [66]. COVID-19 patients show a broad spectrum of endothelial alterations, such as an increase in the activity of coagulation factor VIII and a high increase in von Willebrand factor [67] and angiotensin II level in the plasma, associated with viral load and lung damage [68]. Angiotensin II leads to microvascular permeability [69], to the transcription of tissue factor in endothelial cells [70] and to activation of platelets [71]. Angiotensin II can trigger the release of several components of the complement system from endothelial cells [72, 73]. These mechanisms support the key role of the endothelium in the pathogenesis of thrombosis in COVID-19 patients [74, 75]. When endothelial damage occurs, extracellular matrix is exposed to circulating blood, favoring platelet binding. Moreover, endothelial lung damage is considered the hallmark of ARDS [76]. In this scenario, the relationship between damaged endothelial cells and impaired platelet function gains impact in COVID-19 disease. In physiological conditions, at least five main mechanisms stabilize the lung endothelial basal barrier function, such as the release of soluble mediators that promote barrier integrity through endothelial signaling; the physical obstruction of the spaces in the endothelial barrier; the maintenance of the main structural characteristics of the endothelial cells necessary for the integrity of the barrier; stimulation or enhancement of endothelial cell growth and the neutralization or elimination of agents that increase endothelial permeability and compromise the integrity of the barrier [77]. Megakaryocytes were reported in samples from Fox et al. and Aguiar et al. [29, 43]. Therefore, megakaryocytes and platelets appear to be reprogrammed under certain disease conditions. In such contexts, the profile of platelet signaling factors that can influence the function and permeability of the endothelial barrier can change from a stabilizing state to a predominantly inflammatory one. Platelets and platelet products, including serotonin and TxA2, have been implicated in the pulmonary vascular blood pressure response to LPS infusion and other experimental models of ALI ARDS, but there are differences in results that may depend on species and other variables [78]. As in systemic circulation, pulmonary vascular reactivity responses to factors released by platelets depend on whether the endothelium is intact and capable of synthesizing vasodilators, including PGI2 and NO [79].

The endothelial cellular damage during COVID-19 is probably induced more by mechanisms of innate immunity with alternative cell death mechanisms to apoptosis that induce inflammation (necroptosis) [80] and immunothrombosis (netosis) [81]. Results from this study enforce evidence coming from other studies where inflammatory response seems to play an important role in lung damage and gravity of symptoms [82–84].

The proposed pathological mechanism, after infection, regards the role of both innate and adaptive components of the immune system. Lingeswaran at al. reviewed the immunological mechanisms undergoing in COVID-19 disease. They report a delayed type-1 INF response in the early phase of disease by innate immune cells, leading to an ineffective T-cell response. Subsequently, macrophages and recruited lymphocytes are responsible of pro-inflammatory mediators secretion. These mechanisms lead to a cytokine storm, contributing to the worsening of the disease [82].

Cross-talk between inflammatory cells and SARS-Cov-2 has been attributed to Toll-like receptors (TLRs). Among this, TLR4 acquired an important role in COVID-19 disease [85]. A key point is the activation of the TLRs both in platelets and in the endothelium, which can be induced both by direct binding of the SARS-CoV-2 and by indirect activation of cellular damage [86]. Choudhury et al. suggest that TLR4 is most likely to bind molecular patterns from SARS-CoV-2 promoting inflammatory pathways [85]. This molecular mechanism could be the crosstalk between the destruction of the vascular endothelial barrier in the pulmonary interstitium with the formation of proteinaceous edema and the activation of microthrombosis. Interestingly, TLR activation modulates the microvascular permeability and expression of coagulation mediators [87]. Both of these factors are important in COVID-19 leading to edematous lung damage and the formation of microthrombi, events that induce the activation of known mechanisms in sepsis and that induce multi-organ failure [88]. Moreover, TLR4 of EC, is able to activate mononuclear cells by the secretion of IL-6 [89]. Virus induced activation of TLR4 may be an important mechanism in COVID-19 disease. This consideration might explain the possible response to drugs modulating IL-6 levels [90]. IL-6 is a highly inducible pro-inflammatory cytokine secreted by several cell types including monocytes, lymphocytes, fibroblasts and endothelial cells; interleukin-1β (IL-1β) and TNF-α, viral infection and Angiotensin II are able to induce IL-6 [91, 92]. IL-6 plays an important role in the activation of endothelial cells during the initial phase of inflammation, inducing greater vascular permeability, the secretion of pro-inflammatory cytokines / chemokines by endothelial cells (IL-6, IL- 8, MCP-1 and complement activation C5a). In COVID-19 patients, IL-6 levels appear to be directly related to disease severity [93, 94]. Another important cytokine with increased serum levels observed in COVID-19 is soluble IL-2R, which is also related to the severity of the disease [95, 96]. Endothelial lung cells have also been shown to express IL-2R on their surface and that IL-2 could bind to endothelial cells and induce pulmonary edema in response to this link [97, 98]. Summarizing, IL-2R expression and IL-2 response may be implicated in the pathophysiology of COVID-19. Finally, pro-inflammatory cytokines, in particular IL-1β, IL-6 and TNFα, which are elevated in patients with COVID-19 induce the loss of normal antithrombotic and anti-inflammatory functions of endothelial cells, leading to dysregulation of coagulation, complement and platelet activation and leukocyte recruitment, explaining the wide range of immunological cells and the wide spectrum of histopathologic alterations. Recently two meta-analysis have been published investigating the administration of IL-6 inhibitors (Tocilizumab) in patients with COVID-19 disease. Improved overall survival resulted in severe patients after Tocilizumab administration [99, 100].

Recently, over the alterations in immunological response, complement and coagulation dysfunction have been associated to a worse outcome in patients with COVID-19 disease [101–103]. Results coming from this systematic review show evidence of microthrombi, hemorrhage and both interstitial and alveolar edema. These pathological processes have occurred in autopsy cases and it has been clinically proven that patients with alterations of coagulation-fibrinolysis parameters (D-dimers, PT, APTT, Fibrinogen, platelet count, FDP, AT, uPA, PAI-1), in particular if associated with parameters of inflammation, cytokine storm, activation of macrophages and endothelial cells (PCR, TNF-alpha, IL-1, IL-6, IL-8, IL-10, ferritin) reported a greater risk of developing severe pathology and to die from the disease [104]. As reported by Polak et al., fibro-proliferative processes were documented also in patients during the pre-symptomatic period and in the early phase of disease [63]. These processes have been already descripted during the early phase of ARDS [105] and an alteration of the balance between coagulation and fibrinolysis regulates pathological cell remodeling during the DAD [106]. Furthermore, coagulation is also part of the early-innate response to infections, in fact, coagulation system has evolved as an effector pathway of the immune response, by depositing fibrin around bacteria, trapping them, preventing their dissemination and favoring innate and specific defenses [107]. In COVID-19 disease, autopsy diagnostic findings demonstrate the presence of microthrombosis with associated perivascular inflammation, showing a possible role of endothelial inflammation in microvascular thrombosis. Although this hypothesis should be evaluated in future studies, there is emerging evidence that in COVID-19 disease, the damage of the endothelium could represent a cardinal event of the prothrombotic state and therefore microthrombosis may occur as primary event related to the SARS-CoV-2 virus infection and to the abnormal innate and adaptive immunity response, highlighting the role of host immunity in the development of COVID-19 disease. Villar et al. define the ability of corticosteroids to decrease the contemporary association between inflammation – coagulation and fibro-proliferation, with consequences on COVID-19 disease resolution [108].

These synergistic processes are histologically associated to DAD, in particular to hyaline membranes and inflammatory exudation [19, 108]. Inflammation, coagulation and fibro-proliferative processes may occur earlier, before the evidence of clinically relevant ARDS. While worsening of disease, the establishing of clinical ARDS, leads to a general status of hypoxia, which may induce an increase of Tissue factor, VIIa factor and Serpin 1, which in turn are primarily responsible for the development of a pro-coagulant and anti-fibrinolytic state [109, 110]. In previously reported animal models of SARS, alterations in coagulation and intra-alveolar deposition of fibrin proceed in parallel and occur in a particularly complex scenario in which pro-inflammatory factors participate (IL-1, TNF-alpha, IL-6), pro-fibrotic (TGF, CTGF, PDGF), together with the increase expression of the urokinetic pathway with activation of both pro-fibrinolytic and anti-fibrinolytic genes. Moreover, factors involved in plasminogen activation determine the activation of the plasminogen-plasmin system and are related to lethality; also the synergistic association between inflammatory factors and expression of fibrinolytic genes can explain the bleeding aspects observed in animal models [111]. This scenario is well described in Menter et al., and Yao et al. COVID-19 autopsy [18, 31], where necrotic-hemorrhagic aspects are observed together with endoalveolar and interstitial fibrosis associated with diffuse microthrombosis. Meanwhile, microthrombosis has not been identified in post-mortem biopsies in a study of Tian et al. [26]. Retrospective clinical evidence, have shown in Chinese patients that the use of low molecular weight heparins is related to a better clinical outcome only in cases with an increase in D-dimers and with alteration of the parameters of the coagulation. Administration of heparin is associated with adverse events when the patient does not present high D-Dimers and when there is no alteration of the coagulation parameters [112, 113]. Contrary to these reported clinical retrospective studies, the results of histopathological studies showed that D-dimers are elevated in almost all fatal cases of Covid-19 disease; furthermore, in a fair proportion of cases (29%) the D-dimers were ten times higher than the normal limits [34, 63]. It seems useful using anticoagulant treatment in severe conditions of COVID-19, in order to reduce mortality [114–116], while, using of prophylactic administration of low molecular weight heparin in early phase of COVID-19 disease has to be ascertained by randomized clinical trial (NCT04492254 – https://clinicaltrials.gov/ct2/show/NCT04492254 - NIH-clinicaltrials.gov) [117], considering also, the non-anticoagulant related effects of heparin [116].

Another histopathological alteration in COVID-19 disease is the evidence of pulmonary fibrosis. Generally, fibrosis can be result of chronic inflammation or can develop as primary fibro-proliferative process, genetically influenced and age-related, in idiopathic pulmonary fibrosis (IPF). Pulmonary fibrosis is a recognized sequel of ARDS. This is characterized by a diffuse alveolar pulmonary epithelial and endothelial damage, leading to an increase in pulmonary edema, permeability and alveolar filling [118]. Numerous epidemiological, viral and current clinical evidences support the possibility that pulmonary fibrosis may be one of the main complications in COVID-19 patients [119, 120]. Fibrosis is result of different mechanisms, which collaborate in promoting fibrin deposition. Damaged alveoli release different markers both in the blood and in the alveolar compartment; contemporary, vascular endothelial injury leads to an increase of microvascular permeability and alveolar edema. In some patients, the significant and persistent accumulation of macrophages, fibrocytes, fibroblasts and myofibroblasts in the alveolar compartment leads to excessive deposition of extracellular matrix components including fibronectin and type I and III collagen, among other proteins. An imbalance between profibrotic and antifibrotic mediators may subsequently determine this fibroproliferative response [121, 122]. The pathophysiological process of pulmonary fibrosis is believed to be an abnormal wound healing state. The abnormal proliferation of fibroblasts and the accumulation of ECM proteins (such as collagen) have become the center of recent research on pulmonary fibrosis [123]. Interestingly, circulating fibroblasts are positively correlated with the degree of fibrosis, indicating which patients with IPF may face an increased risk of unfavorable prognosis. CXCR4 is the main chemokine receptor expressed on circulating fibroblasts in humans and mice, and there is a direct correlation between the lung and plasma levels of CXCL12 and the number of circulating pulmonary fibrocytes in patients with pulmonary fibrosis [124].

The rationale for the use of antifibrotic therapy is based on the spectrum of fibrotic lung disease observed in COVID-19, which ranges from fibrosis associated with the organization of pneumonia to severe ALI, in which there is evolution to widespread fibrotic change [125]. However, these drugs do not address the immune dysregulation of SARS-CoV-2 infection, nor they are able to attenuate the prothrombotic aspects of this complex pathogenic process [119]. Although these considerations, an interesting target for antifibrotic therapies can be the TGF-β pathway. There are drugs in development that target various molecules in this pathway, including anti-integrin αvβ6, PLN-74809 and galectins. These are particularly interesting candidates because the SARS-CoV-2 spike protein contains an Arg-Gly-Asp integrin-binding domain and a number of coronaviruses contain an N-terminal galectin fold [126]. Strategies for blocking αvβ6 integrin have been evaluated in vivo models of ALI [127, 128]. Furthermore, IL-1, which has been identified as a key component of the cytokine storm in COVID-19 and other viruses, could mediate its effects through Arg-Gly-Asp binding integrin [129]. Other studies have identified mTOR as an emerging target in IPF [130, 131]. In addition, PRM-151 is an analogue of SAP (also known as PTX2), which is a member of the pentraxin family of proteins that includes C-reactive protein and PTX3 and has shown promise in a phase 2 study for IPF [132].

In conclusion, COVID-19 lethal cases appear as a heterogeneous disease, characterized by of contemporary presence of different histological findings, which reflect diverse pathological pathways. Epithelial, vascular and fibrotic changes may occur as separate alterations or together (Fig. 2). Histological evidence of edema, together with DAD and events of microthrombosis in major of deceased COVID-19 patients, suggest the use of combined and early administration of steroid-centered therapy, in association of targeted therapies. For example, promising results come from a single center observational study, in which the association of Tocilizumab and steroid was associated with better outcome [133]. Future studies should detect reliable biomarkers of lung tissue pathological status, in order to develop target therapies.

## Supplementary Information

**Additional file 1: Supplemental Table 1** List of excluded studies and reason of exclusion.

## Data Availability

Not applicable.
