# Supplementary material for: Lung histopathological findings in COVID-19 disease – a systematic review
Source: Infect Agent Cancer. 2021 May 17;16:34. doi: 10.1186/s13027-021-00369-0 (PMC8127295; doi:10.1186/s13027-021-00369-0)
Supplement: Supplementary file 1 — Additional file 1: Supplemental Table 1 List of excluded studies and reason of exclusion. [file 13027_2021_369_MOESM1_ESM.docx]

| **Author and Year** | **Reason for exclusion** |
| --- | --- |
| Wang et al. (2020) (1) | Article in chinese and review |
| Xie et al. (2020) (2) | Review |
| Liu et al. (2020) (3) | Article in chinese |
| Asadi-Pooya et al. (2020) (4) | Not dealing with pulmonary tissue |
| Wang et al. (2020) (5) | Article in chinese |
| Von der Thusen et al. (2020) (6) | Unascertained exposure |
| Fuk-Woo Chan et al. (2020) (7) | Animal model |
| Barnes et al. (2020) (8) | Review |
| Yao et al. (2020) (9) | Article in chinese |
| Mason (2020) (10) | Not in human study |
| Rockx et al. (2020) (11) | Not in human study |
| Ding et al. (2020) (12) | Article in chinese |
| Solaimanzadeh (2020) (13) | No histological data |
| Jain (2020) (14) | No Sars-Cov2 infection |
| Li et al. (2020) (15) | Review |
| Mao et al. (2020) (16) | Article in chinese |
| Chen (2020) (17) | Article in chinese |
| Xiao et al. (2020) (18) | Not dealing with pulmonary tissue |
| Prasad et al. (2020) (19) | No tissue analized |
| Chen et al. (2020) (20) | Article in chinese |
| Zhang et al. (2020) (21) | Article in chinese |
| Wang (2020) (22) | Article in chinese |
| Hotez et al. (2020) (23) | No histological data |
| Su et al. (2020) (24) | Not dealing with pulmonary tissue |
| Sungnak et al. (2020) (25) | No histological data |
| Cong (2020) (26) | Article in chinese |
| Liu et al. (2020) (27) | Article in chinese |
| Zhu et al.(2020) (28) | No histological data |
| Huang et al. (2020) (29) | No histological data |
| Chen et al. (2020) (30) | Article in chinese |
| Gianotti et al. (2020) (31) | Not dealing with pulmonary tissue |
| Xu et al. (2020) (32) | Article in chinese |
| Lagana et al. (2020) (33) | Not dealing with pulmonary tissue |
| Sala et al. (2020) (34) | Not dealing with pulmonary tissue |
| Giani et al. (2020) (35) | No histological data |
| Paniz-Mondolfi et al. (2020) (36) | Not dealing with pulmonary tissue |
| Guerini-Rocco et al. (2020) (37) | Not dealing with pulmonary tissue |
| Mulvey et al. (2020) (38) | Not dealing with pulmonary tissue |
| Barth et al. (2020) (39) | No histological data |
| Xu et al. (2020) (40) | Article in chinese |
| Farkash et al. (2020) (41) | Not dealing with pulmonary tissue |
| Peleg et al. (2020) (42) | Not dealing with pulmonary tissue |
| Kuang et al. (2020) (43) | Article in chinese |
| Nasr et al. (2020) (44) | Not dealing with pulmonary tissue |
| Tian et al. (2020) (45) | No histological data |
| Colonna et al. (2020) (46) | Not dealing with pulmonary tissue |
| Baud et al. (2020) (47) | Not dealing with pulmonary tissue |
| Kolivras et al. (2020) (48) | Not dealing with pulmonary tissue |
| Liu et al. (2020) (49) | No histological data |
| Binder et al. (2020) (50) | Not dealing with pulmonary tissue |
| Locatelli et al. (2020) (51) | Not dealing with pulmonary tissue |
| Jimenez-Cauhe et al. (2020) (52) | Not dealing with pulmonary tissue |
| Fernandez-Nieto et al. (2020) (53) | Not dealing with pulmonary tissue |
| Herrero-Moyano et al. (2020) (54) | Not dealing with pulmonary tissue |
| Deshpande (2020) (55) | Review |
| Shanes et al. (2020) (56) | Not dealing with pulmonary tissue |
| Llamas-Velasco et al. (2020) (57) | Not dealing with pulmonary tissue |
| Wang et al. (2020) (58) | Not dealing with pulmonary tissue |
| Garcia-Gil et al. (2020) (59) | Not dealing with pulmonary tissue |
| Reichard et al.(2020) (60) | Not dealing with pulmonary tissue |
| Torrelo et al. (2020) (61) | Not dealing with pulmonary tissue |
| El Hachem et al. (2020) (62) | Not dealing with pulmonary tissue |
| Ignat et al. (2020) (63) | Not dealing with pulmonary tissue |
| Kissling et al. (2020) (64) | Not dealing with pulmonary tissue |
| Cantu et al. (2020) (65) | No histological data |
| Lv et al. (2020) (66) | Not dealing with pulmonary tissue |
| Antinori et al. (2020) (67) | No histological data |
| Bhayana et al. (2020) (68) | No dealing with pulmonary tissue |
| Stadlmann et al. (2020) (69) | No tissue involved |
| Gianotti et al. (2020) (70) | Not dealing with pulmonary tissue |
| Joob et al. (2020) (71) | No histological data |

REFERENCES

1. Wang HJ, Du SH, Yue X, Chen CX. Review and Prospect of Pathological Features of Corona Virus Disease. Fa Yi Xue Za Zhi. 2020;36(1):16-20.

2. Xie M, Chen Q. Insight into 2019 novel coronavirus - An updated interim review and lessons from SARS-CoV and MERS-CoV. Int J Infect Dis. 2020;94:119-24.

3. Liu Q, Wang RS, Qu GQ, Wang YY, Liu P, Zhu YZ, et al. Gross examination report of a COVID-19 death autopsy. Fa Yi Xue Za Zhi. 2020;36(1):21-3.

4. Asadi-Pooya AA, Simani L. Central nervous system manifestations of COVID-19: A systematic review. J Neurol Sci. 2020;413:116832.

5. Wang X, Ding YQ. [From SARS to COVID-19: pathogens, receptor, pathogenesis and principles of the treatment]. Zhonghua Bing Li Xue Za Zhi. 2020;49(6):647-52.

6. von der Thusen J, van der Eerden M. Histopathology and genetic susceptibility in COVID-19 pneumonia. Eur J Clin Invest. 2020:e13259.

7. Chan JF, Zhang AJ, Yuan S, Poon VK, Chan CC, Lee AC, et al. Simulation of the clinical and pathological manifestations of Coronavirus Disease 2019 (COVID-19) in golden Syrian hamster model: implications for disease pathogenesis and transmissibility. Clin Infect Dis. 2020.

8. Barnes BJ, Adrover JM, Baxter-Stoltzfus A, Borczuk A, Cools-Lartigue J, Crawford JM, et al. Targeting potential drivers of COVID-19: Neutrophil extracellular traps. J Exp Med. 2020;217(6).

9. Yao XH, Li TY, He ZC, Ping YF, Liu HW, Yu SC, et al. [A pathological report of three COVID-19 cases by minimal invasive autopsies]. Zhonghua Bing Li Xue Za Zhi. 2020;49(5):411-7.

10. Mason RJ. Pathogenesis of COVID-19 from a cell biology perspective. Eur Respir J. 2020;55(4).

11. Rockx B, Kuiken T, Herfst S, Bestebroer T, Lamers MM, Oude Munnink BB, et al. Comparative pathogenesis of COVID-19, MERS, and SARS in a nonhuman primate model. Science. 2020;368(6494):1012-5.

12. Ding YQ, Bian XW. [Analysis of coronavirus disease-19 (COVID-19) based on SARS autopsy]. Zhonghua Bing Li Xue Za Zhi. 2020;49(4):291-3.

13. Solaimanzadeh I. Acetazolamide, Nifedipine and Phosphodiesterase Inhibitors: Rationale for Their Utilization as Adjunctive Countermeasures in the Treatment of Coronavirus Disease 2019 (COVID-19). Cureus. 2020;12(3):e7343.

14. Jain A. COVID-19 and lung pathology. Indian J Pathol Microbiol. 2020;63(2):171-2.

15. Li H, Liu Z, Ge J. Scientific research progress of COVID-19/SARS-CoV-2 in the first five months. J Cell Mol Med. 2020;24(12):6558-70.

16. Mao DM, Zhou N, Zheng D, Yue JC, Zhao QH, Luo B, et al. Guide to the Forensic Pathology Practice on Death Cases Related to Corona Virus Disease 2019 COVID-19 Trial Draft. Fa Yi Xue Za Zhi. 2020;36(1):6-5.

17. Chen J. [How to understand the histopathology of SARS and coronavirus disease-19 (COVID-19) associated with acute respiratory distress syndrome]. Zhonghua Bing Li Xue Za Zhi. 2020;49(4):289-90.

18. Xiao F, Tang M, Zheng X, Liu Y, Li X, Shan H. Evidence for Gastrointestinal Infection of SARS-CoV-2. Gastroenterology. 2020;158(6):1831-3 e3.

19. Prasad S, Potdar V, Cherian S, Abraham P, Basu A, Team I-NN. Transmission electron microscopy imaging of SARS-CoV-2. Indian J Med Res. 2020;151(2 & 3):241-3.

20. Chen S, Huang B, Luo DJ, Li X, Yang F, Zhao Y, et al. [Pregnancy with new coronavirus infection: clinical characteristics and placental pathological analysis of three cases]. Zhonghua Bing Li Xue Za Zhi. 2020;49(5):418-23.

21. Zhang T, Sun LX, Feng RE. [Comparison of clinical and pathological features between severe acute respiratory syndrome and coronavirus disease 2019]. Zhonghua Jie He He Hu Xi Za Zhi. 2020;43(6):496-502.

22. Wang HJ. From SARS-CoV to SARS-CoV-2: The response and challenge of forensic infectious disease autopsy. Fa Yi Xue Za Zhi. 2020;36(1):1-3.

23. Hotez PJ, Bottazzi ME, Corry DB. The potential role of Th17 immune responses in coronavirus immunopathology and vaccine-induced immune enhancement. Microbes Infect. 2020;22(4-5):165-7.

24. Su H, Yang M, Wan C, Yi LX, Tang F, Zhu HY, et al. Renal histopathological analysis of 26 postmortem findings of patients with COVID-19 in China. Kidney Int. 2020;98(1):219-27.

25. Sungnak W, Huang N, Becavin C, Berg M, Queen R, Litvinukova M, et al. SARS-CoV-2 entry factors are highly expressed in nasal epithelial cells together with innate immune genes. Nat Med. 2020;26(5):681-7.

26. Cong B. Academician Cong Bin: Autopsy of SARS-CoV-2 infection is needed to be strengthened. Fa Yi Xue Za Zhi. 2020;36(1):4-5.

27. Liu M, Feng RE, Li Q, Zhang HK, Wang YG. [Comparison of pathological changes and pathogenic mechanisms caused by H1N1 influenza virus, highly pathogenic H5N1 avian influenza virus, SARS-CoV, MERS-CoV and 2019-nCoV]. Zhonghua Bing Li Xue Za Zhi. 2020;49(5):511-6.

28. Zhu N, Zhang D, Wang W, Li X, Yang B, Song J, et al. A Novel Coronavirus from Patients with Pneumonia in China, 2019. N Engl J Med. 2020;382(8):727-33.

29. Huang Z, Zhao S, Xu L, Chen J, Lin W, Zeng H, et al. Imaging features and mechanisms of novel coronavirus pneumonia (COVID-19): Study Protocol Clinical Trial (SPIRIT Compliant). Medicine (Baltimore). 2020;99(16):e19900.

30. Chen XB, Du SH, Lu JC, Tan XH, Li DR, Yue X, et al. Retrospective Analysis of 61 Cases of Children Died of Viral Pneumonia. Fa Yi Xue Za Zhi. 2020;36(2):164-8.

31. Gianotti R, Veraldi S, Recalcati S, Cusini M, Ghislanzoni M, Boggio F, et al. Cutaneous Clinico-Pathological Findings in three COVID-19-Positive Patients Observed in the Metropolitan Area of Milan, Italy. Acta Derm Venereol. 2020;100(8):adv00124.

32. Xu SP, Kuang D, Hu Y, Liu C, Duan YQ, Wang GP. [Detection of 2019-nCoV in the pathological paraffin embedded tissue]. Zhonghua Bing Li Xue Za Zhi. 2020;49(4):354-7.

33. Lagana SM, De Michele S, Lee MJ, Emond JC, Griesemer AD, Tulin-Silver SA, et al. COVID-19 Associated Hepatitis Complicating Recent Living Donor Liver Transplantation. Arch Pathol Lab Med. 2020.

34. Sala S, Peretto G, Gramegna M, Palmisano A, Villatore A, Vignale D, et al. Acute myocarditis presenting as a reverse Tako-Tsubo syndrome in a patient with SARS-CoV-2 respiratory infection. Eur Heart J. 2020;41(19):1861-2.

35. Giani M, Seminati D, Lucchini A, Foti G, Pagni F. Exuberant Plasmocytosis in Bronchoalveolar Lavage Specimen of the First Patient Requiring Extracorporeal Membrane Oxygenation for SARS-CoV-2 in Europe. J Thorac Oncol. 2020;15(5):e65-e6.

36. Paniz-Mondolfi A, Bryce C, Grimes Z, Gordon RE, Reidy J, Lednicky J, et al. Central nervous system involvement by severe acute respiratory syndrome coronavirus-2 (SARS-CoV-2). J Med Virol. 2020;92(7):699-702.

37. Guerini-Rocco E, Taormina SV, Vacirca D, Ranghiero A, Rappa A, Fumagalli C, et al. SARS-CoV-2 detection in formalin-fixed paraffin-embedded tissue specimens from surgical resection of tongue squamous cell carcinoma. J Clin Pathol. 2020.

38. Mulvey JJ, Magro CM, Ma LX, Nuovo GJ, Baergen RN. Analysis of complement deposition and viral RNA in placentas of COVID-19 patients. Ann Diagn Pathol. 2020;46:151530.

39. Barth RF, Xu X, Buja LM. A Call to Action: The Need for Autopsies to Determine the Full Extent of Organ Involvement Associated With COVID-19. Chest. 2020;158(1):43-4.

40. Xu X, Chang XN, Pan HX, Su H, Huang B, Yang M, et al. [Pathological changes of the spleen in ten patients with coronavirus disease 2019(COVID-19) by postmortem needle autopsy]. Zhonghua Bing Li Xue Za Zhi. 2020;49(6):576-82.

41. Farkash EA, Wilson AM, Jentzen JM. Ultrastructural Evidence for Direct Renal Infection with SARS-CoV-2. J Am Soc Nephrol. 2020.

42. Peleg Y, Kudose S, D'Agati V, Siddall E, Ahmad S, Kisselev S, et al. Acute Kidney Injury Due to Collapsing Glomerulopathy Following COVID-19 Infection. Kidney Int Rep. 2020.

43. Kuang D, Xu SP, Hu Y, Liu C, Duan YQ, Wang GP. [Pathological changes with novel coronavirus infection in lung cancer surgical specimen]. Zhonghua Bing Li Xue Za Zhi. 2020;49(5):471-3.

44. Nasr SH, Kopp JB. COVID-19-Associated Collapsing Glomerulopathy: An Emerging Entity. Kidney Int Rep. 2020.

45. Tian S, Xiao SY. Pathology of 2019 Novel Coronavirus Pneumonia: A Dynamic Disease Process. J Thorac Oncol. 2020;15(5):e67-e8.

46. Colonna C, Monzani NA, Rocchi A, Gianotti R, Boggio F, Gelmetti C. Chilblain-like lesions in children following suspected COVID-19 infection. Pediatr Dermatol. 2020;37(3):437-40.

47. Baud D, Greub G, Favre G, Gengler C, Jaton K, Dubruc E, et al. Second-Trimester Miscarriage in a Pregnant Woman With SARS-CoV-2 Infection. JAMA. 2020;323(21):2198-200.

48. Kolivras A, Dehavay F, Delplace D, Feoli F, Meiers I, Milone L, et al. Coronavirus (COVID-19) infection-induced chilblains: A case report with histopathologic findings. JAAD Case Rep. 2020.

49. Liu J, Babka AM, Kearney BJ, Radoshitzky SR, Kuhn JH, Zeng X. Molecular detection of SARS-CoV-2 in formalin-fixed, paraffin-embedded specimens. JCI Insight. 2020;5(12).

50. Binder L, Hogenauer C, Langner C. Gastrointestinal effects of an attempt to avoid contracting COVID-19 by 'disinfection'. Histopathology. 2020.

51. Locatelli AG, Robustelli Test E, Vezzoli P, Carugno A, Moggio E, Consonni L, et al. Histologic features of long-lasting chilblain-like lesions in a paediatric COVID-19 patient. J Eur Acad Dermatol Venereol. 2020.

52. Jimenez-Cauhe J, Ortega-Quijano D, Carretero-Barrio I, Suarez-Valle A, Saceda-Corralo D, Moreno-Garcia Del Real C, et al. Erythema multiforme-like eruption in patients with COVID-19 infection: clinical and histological findings. Clin Exp Dermatol. 2020.

53. Fernandez-Nieto D, Ortega-Quijano D, Jimenez-Cauhe J, Burgos-Blasco P, de Perosanz-Lobo D, Suarez-Valle A, et al. Clinical and histological characterization of vesicular COVID-19 rashes: a prospective study in a tertiary care hospital. Clin Exp Dermatol. 2020.

54. Herrero-Moyano M, Capusan TM, Andreu-Barasoain M, Alcantara-Gonzalez J, Ruano-Del Salado M, Sanchez-Largo Uceda ME, et al. A clinicopathological study of eight patients with COVID-19 pneumonia and a late-onset exanthema. J Eur Acad Dermatol Venereol. 2020.

55. Deshpande C. Thromboembolic Findings in COVID-19 Autopsies: Pulmonary Thrombosis or Embolism? Ann Intern Med. 2020;173(5):394-5.

56. Shanes ED, Mithal LB, Otero S, Azad HA, Miller ES, Goldstein JA. Placental Pathology in COVID-19. Am J Clin Pathol. 2020;154(1):23-32.

57. Llamas-Velasco M, Munoz-Hernandez P, Lazaro-Gonzalez J, Reolid-Perez A, Abad-Santamaria B, Fraga J, et al. Thrombotic occlusive vasculopathy in a skin biopsy from a livedoid lesion of a patient with COVID-19. Br J Dermatol. 2020.

58. Wang Y, Liu S, Liu H, Li W, Lin F, Jiang L, et al. SARS-CoV-2 infection of the liver directly contributes to hepatic impairment in patients with COVID-19. J Hepatol. 2020.

59. Garcia-Gil MF, Garcia Garcia M, Monte Serrano J, Prieto-Torres L, Ara-Martin M. Acral purpuric lesions (erythema multiforme type) associated with thrombotic vasculopathy in a child during the COVID-19 pandemic. J Eur Acad Dermatol Venereol. 2020.

60. Reichard RR, Kashani KB, Boire NA, Constantopoulos E, Guo Y, Lucchinetti CF. Neuropathology of COVID-19: a spectrum of vascular and acute disseminated encephalomyelitis (ADEM)-like pathology. Acta Neuropathol. 2020;140(1):1-6.

61. Torrelo A, Andina D, Santonja C, Noguera-Morel L, Bascuas-Arribas M, Gaitero-Tristan J, et al. Erythema multiforme-like lesions in children and COVID-19. Pediatr Dermatol. 2020;37(3):442-6.

62. El Hachem M, Diociaiuti A, Concato C, Carsetti R, Carnevale C, Ciofi Degli Atti M, et al. A clinical, histopathological and laboratory study of 19 consecutive Italian paediatric patients with chilblain-like lesions: lights and shadows on the relationship with COVID-19 infection. J Eur Acad Dermatol Venereol. 2020.

63. Ignat M, Philouze G, Aussenac-Belle L, Faucher V, Collange O, Mutter D, et al. Small bowel ischemia and SARS-CoV-2 infection: an underdiagnosed distinct clinical entity. Surgery. 2020;168(1):14-6.

64. Kissling S, Rotman S, Gerber C, Halfon M, Lamoth F, Comte D, et al. Collapsing glomerulopathy in a COVID-19 patient. Kidney Int. 2020;98(1):228-31.

65. Cantu MD, Towne WS, Emmons FN, Mostyka M, Borczuk A, Salvatore SP, et al. Clinical significance of blue-green neutrophil and monocyte cytoplasmic inclusions in SARS-CoV-2 positive critically ill patients. Br J Haematol. 2020;190(2):e89-e92.

66. Lv D, Xu Y, Cheng H, Ke Y, Zhang X, Ying K. A novel cell-based assay for dynamically detecting neutrophil extracellular traps-induced lung epithelial injuries. Exp Cell Res. 2020:112101.

67. Antinori S, Rech R, Galimberti L, Castelli A, Angeli E, Fossali T, et al. Invasive pulmonary aspergillosis complicating SARS-CoV-2 pneumonia: A diagnostic challenge. Travel Med Infect Dis. 2020:101752.

68. Bhayana R, Som A, Li MD, Carey DE, Anderson MA, Blake MA, et al. Abdominal Imaging Findings in COVID-19: Preliminary Observations. Radiology. 2020:201908.

69. Stadlmann S, Hein-Kuhnt R, Singer G. Viropathic multinuclear syncytial giant cells in bronchial fluid from a patient with COVID-19. J Clin Pathol. 2020.

70. Gianotti R, Zerbi P, Dodiuk-Gad RP. Clinical and histopathological study of skin dermatoses in patients affected by COVID-19 infection in the Northern part of Italy. J Dermatol Sci. 2020;98(2):141-3.

71. Joob B, Wiwanitkit V. Pulmonary Pathology of Early Phase 2019 Novel Coronavirus Pneumonia. J Thorac Oncol. 2020;15(5):e67.
